# Oxidative stress at telomeres triggers internal DNA loops, TRF1 dissociation, and TRF2-dependent R-loops

**DOI:** 10.1093/nar/gkaf285

**Published:** 2025-04-12

**Authors:** Trang Thu Nguyen, Giulia Mazzucco, Eftychia Kyriacou, Thomas Lunardi, Leona Brandl, Wareed Ahmed, Ylli Doksani, Joachim Lingner

**Affiliations:** Swiss Institute for Experimental Cancer Research (ISREC), School of Life Sciences, École Polytechnique Fédérale de Lausanne (EPFL), 1015 Lausanne, Switzerland; IFOM ETS - The AIRC Institute of Molecular Oncology, 20139 Milan, Italy; Swiss Institute for Experimental Cancer Research (ISREC), School of Life Sciences, École Polytechnique Fédérale de Lausanne (EPFL), 1015 Lausanne, Switzerland; Swiss Institute for Experimental Cancer Research (ISREC), School of Life Sciences, École Polytechnique Fédérale de Lausanne (EPFL), 1015 Lausanne, Switzerland; Swiss Institute for Experimental Cancer Research (ISREC), School of Life Sciences, École Polytechnique Fédérale de Lausanne (EPFL), 1015 Lausanne, Switzerland; Swiss Institute for Experimental Cancer Research (ISREC), School of Life Sciences, École Polytechnique Fédérale de Lausanne (EPFL), 1015 Lausanne, Switzerland; IFOM ETS - The AIRC Institute of Molecular Oncology, 20139 Milan, Italy; Swiss Institute for Experimental Cancer Research (ISREC), School of Life Sciences, École Polytechnique Fédérale de Lausanne (EPFL), 1015 Lausanne, Switzerland

## Abstract

Telomeres are the nucleoprotein structures at chromosome ends. Telomeres are particularly sensitive to oxidative stress, which can induce telomere damage, shortening, and premature cellular senescence. How oxidative damage influences telomere structure has not been defined. Here, we induce oxidative damage at telomeres using menadione, which damages mitochondria mimicking intrinsic oxidative stress. We find that oxidative stress induces at telomeres single-stranded DNA breaks, internal DNA loop structures, dissociation of the shelterin component TRF1, upregulation of TERRA long noncoding RNA, and increased DNA:RNA hybrid structures known as R-loops. R-loop formation is enhanced not only in *cis* at telomeres, which show increased TERRA transcription, but also in trans at telomeres at which TERRA transcription is not induced indicating post-transcriptional R-loop formation. Finally, we show that oxidative damage induced R-loop formation requires TRF2, whose R-loop promoting activity may be unleashed upon TRF1 dissociation from telomeres. Altogether, our findings uncover in response to oxidative stress major remodelling of telomeric DNA, RNA, and shelterin complexes, and they unravel a physiological role of TRF2’s ability to stimulate TERRA R-loop formation. We propose that the identified structural changes may facilitate DNA damage signalling and repair pathways to maintain telomere integrity during development and aging.

## Graphical Abstract

**Graphical Abstract:**
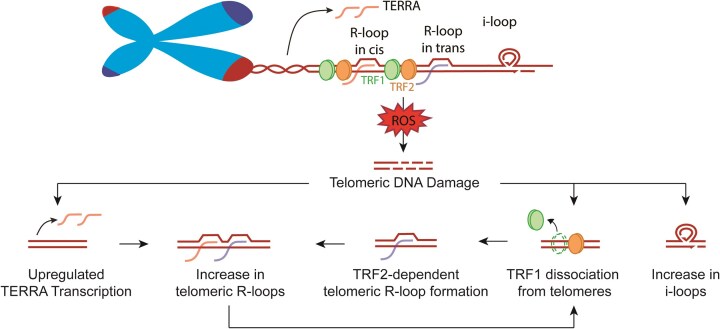

## Introduction

Oxidative stress has been implicated in aging and multiple human diseases including neurodegenerative disorders and cancer [1, 2]. Reactive oxygen species (ROS) are generated by exogenous sources such as UV light, ionizing radiation, and chemicals. Leakage from mitochondria of ROS, which are produced during oxidative phosphorylation, is a major endogenous source of oxidative stress. Telomeres are thought to be particularly vulnerable to oxidative damage, probably due to the presence of a triple-G sequence that is readily modified by ROS in 8-oxoG and the presence of the 3′-single-stranded overhang where base excision repair is inefficient [3, 4]. Oxidized telomeric DNA and oxidized nucleotide precursors also inhibit telomerase activity, which may impact on telomere maintenance in stem cells during aging and in cancer cells that reactivate telomerase [5–7]. However, despite the repression of the DNA damage response at functional telomeres, DNA repair proteins associate with telomeres when exposed to oxidative stress [8]. In addition, antioxidant enzymes including PRDX1 are enriched in telomeric chromatin to locally neutralize ROS species [5].

Telomeres are bound by the shelterin proteins comprising six polypeptides in humans [9]. TRF1 and TRF2 bind as homodimers to the double-stranded telomeric 5′-TTAGGG-3′/5′-CCCTAA-3′ telomeric repeats. POT1 binds to single-stranded 5′-TTAGGG-3′ repeats, which are present as 3′ overhangs at chromosome ends. RAP1, TIN2, and TPP1 bind telomeres indirectly through their physical interaction, respectively, with TRF2, TRF1, and TRF2 and TIN2. Shelterin proteins are crucial for most telomeric functions including suppression of the DNA damage response and telomere length regulation. In addition, telomeres are transcribed into the long noncoding RNA TERRA [10]. TERRA transcription is driven from subtelomeric promoters and proceeds into the telomeric tracts [11–14]. Thus, TERRA contains subtelomere-derived sequences as well as 5′-UUAGGG-3′ telomeric repeats at its 3′ end. TERRA can invade telomeric DNA, forming DNA:RNA hybrid structures in which the G-rich telomeric DNA strand is displaced [15]. These structures are known as R-loops [16]. Post-transcriptional R-loop formation is dependent on the RAD51 DNA recombinase, which also can catalyze R-loop formation *in vitro* [15]. Similarly, the RAD51 interacting protein RAD51AP1 has been implicated in posttranscriptional R-loop formation [17, 18]. In addition, the shelterin protein TRF2 can promote TERRA R-loops [19]. Telomeric R-loops can induce replication stress when present during the S phase of the cell cycle but they can also promote telomeric DNA repair and telomere elongation in pre-senescent cells as well as ALT-cancer cells, which counteract telomere shortening by recombination instead of telomerase [20–23]. Other TERRA functions include its participation in the telomeric DNA damage response [13, 24] and its roles as cell death triggering signaling molecule during carcinogenesis, when telomeres enter replicative crisis ([25]; reviewed in [26]).

Here, we explore the effects of ROS on telomeric DNA structure, shelterin composition, and TERRA. We find that ROS-induced telomeric DNA damage promotes formation of internal loop (i-loop) structures [27]. The telomeric shelterin protein TRF1 dissociates from telomeres, and TERRA transcription increases at some but not all telomeres. Furthermore, the abundance of TERRA R-loops levels at telomeres is dependent on TRF2, whose R-loop promoting activity is suppressed at intact telomeres by TRF1. Altogether, we have found that human cells respond to ROS by inducing a non-anticipated major remodeling of telomeric chromatin structure and composition.

## Materials and methods

### Cell culture

Suspension HEK293E cells were cultured in EX-CELL 293 Serum-Free Medium (Merck) supplemented with 4 mM GlutaMAX. HeLa cells were maintained in Dulbecco’s modified Eagle’s medium (DMEM) (Gibco) supplemented with 100 U/ml of penicillin/streptomycin and 10% fetal bovine serum (FBS) (complete DMEM). Cells were maintained in a controlled humidified atmosphere with 5% (*v*/*v*) CO_2_ at 37°C with constant agitation for suspension HEK293E cells. The generation of HEK293E cells containing endogenously tagged TRF1 and FLAG-TRF2 were described previously [28]. HeLa cells containing endogenously-tagged mCherry-TRF1 were generated in a similar manner. The presence of the mCherry tag was confirmed by western blotting. For oxidative stress induction, cells were treated with 0.1 mg/ml menadione for 2 h with or without prior addition of 5 nM N-acetyl cysteine (NAC) (Sigma–Aldrich) for 30 min. Alternatively, cells were treated with 10 mM H_2_O_2_ (Sigma–Aldrich) for 2 h. After the treatment, cells were washed with cold 1× phosphate-buffered saline (PBS) three times before proceeding with any experiment.

### Small interfering RNA (siRNA) and plasmid transfection

For siRNA transfection, 180 000 cells per well were plated in six-well plates with antibiotic-free DMEM supplemented with 10% FBS 1 day before transfection. The following day, cells were transfected with 20 pmol of siRNA (see Supplementary Table S1) using calcium phosphate transfection. The cells were split 1 day after transfection in complete DMEM and used 2 days after for subsequent experiments.

For short hairpin RNA (shRNA) transfection, 3 × 10^6^ cells per dish were plated in 10-cm dishes with complete DMEM 1 day before transfection. The following day, cells were transfected with Lipofectamine 2000 (Invitrogen) using 20 μg of plasmid DNA (see Supplementary Table S2), following manufacturer’s instructions. The cells were split and selected 1 day after transfection in complete DMEM supplemented with 1 μg/ml of puromycin and used 2 days later for subsequent experiments.

### DNA isolation

Pellets containing 3 × 10^6^ cells were lysed overnight at 37°C in 1 ml of TENS buffer (10 mM Tris–HCl, pH 7.5, 10 mM ethylenediaminetetraacetic acid (EDTA), 100 mM NaCl, 1% sodium dodecyl sulfate (SDS)) supplemented with 100 μg/ml proteinase K. The lysates were pipetted into 15-ml MaXtract High Density tubes (Qiagen) and extracted twice with one volume of Phenol–Chloroform–Isoamyl (PCI) (25:24:1) (Biosolve) pre-warmed to room temperature. The tubes were mixed on a wheel for 10 min and then centrifuged for 5 min at 1500× *g*. The top layers were transferred to new 15 ml tubes, sodium acetate (pH 5.3) was added to 0.3 M, and the DNA was precipitated with one volume of isopropanol. The DNA was collected by centrifugation at 10 000× *g* for 30 min at 4°C, washed twice with 70% ethanol, dried and dissolved in 200 μl of 10 mM Tris–HCl, pH 7.5 overnight at 4°C. The next day, contaminant RNA was digested with 100 ng/μl RNase A (Millipore) for 30 min at 37°C, followed by RNase A inactivation by 100 ng/μl proteinase K treatment for 1 h at 50°C. The DNA was purified with PCI as described above and dissolved in 100 μl of 10 mM Tris–HCl, pH 7.5 overnight at 4°C. DNA concentration and purity were measured on a NanoDrop Spectrophotometer (Thermo Fisher Scientific). The DNA was then used for alkaline or native gel electrophoresis.

### Gel electrophoresis

Six micrograms of genomic DNA were digested with HinfI and RsaI (New England Biolabs (NEB)) (10 U each) in a 50-μl reaction containing 1× CutSmart buffer (NEB) overnight at 37°C. For single-stranded break (SSB) analysis, 2 μg of digested DNA was separated at 2 V/cm for 18 h on a 0.8% alkaline agarose gel (50 mM NaOH, 1 mM EDTA pH 8.0). The gel was then neutralized with neutralization buffer (0.5 M Tris–HCl, pH 7.5, 1.5 M NaCl) for 1 h and dried for 2.5 h at 55°C on a gel dryer (Bio-Rad). For double-stranded break analysis, we carried out native gel electrophoresis in which 2 μg of digested DNA was separated on 0.8% agarose gels in 1× Tris-borate-EDTA (TBE) and dried, as described above. The dried gels were soaked in denaturation buffer (0.5 M NaOH, 1.5 M NaCl) for 1 h followed by neutralization buffer for 1 h. Alkaline and native gels were both pre-hybridized with Church buffer (0.5 M NaHPO_4_, 1 mM EDTA, pH 8.0, 1% (*w*/*v*) bovine serum albumin (BSA), 7% SDS) for at least 1 h, followed by overnight incubation with radiolabeled telomere-specific probes at 50°C. After hybridization, the gels were washed once with 4× saline-sodium citrate (SSC), twice with 4× SSC, 0.1% SDS, twice with 2× SSC, 0.1% SDS, and once with 4× SSC for at least 30 min each at 50°C, and then exposed to a phosphor imager screen overnight. The screen was scanned using a Typhoon imager (GE). Average telomeric DNA fragment length was estimated based on densitometry using AIDA software. The quantification of DNA damage was represented by the percentage decrease in telomere length of treated samples compared to untreated samples.

### Telomeric DNA enrichment

The enrichment procedure was performed as described in [29], with some modifications. Approximately 500 × 10^6^ cells were used for each enrichment. The cells were treated four consecutive times with trioxsalen and UV irradiation. Nuclei were collected by lysing the cells with ice-cold RLN buffer for 5 min, followed by centrifugation at 300× *g* for 2 min at 4°C. The nuclei were lysed with 40 ml of TNES buffer (10 mM Tris–HCl, pH 8.0, 100 mM NaCl, 10 mM EDTA, 0.5% SDS) supplemented with 100 μg/ml proteinase K (Roche) overnight at 37°C. Total DNA was purified by extraction with PCI, precipitated with isopropanol in presence of 0.3 M sodium acetate (pH 5.3), and dissolved in 2 ml of 10 mM Tris–HCl, pH 8.0 overnight at 4°C. DNA concentration was measured using the Qubit Broad Range assay kit (Invitrogen) following the manufacturer’s instructions.

Telomeric DNA was enriched following the published protocol [29]. Upon digestion of 2 mg of genomic DNA with HinfI and MspI, the DNA was first fractionated by a sucrose gradient centrifugation. The enriched telomeric DNA was then digested with HinfI, MspI, RsaI, AluI, MboI, HphI, and MnlI, and the digestion-resistant large telomeric DNA fragments were further purified on agarose gels as a second step. The telomeric DNA was eluted in TE buffer and quantified using the Qubit High Sensitivity assay kit (Invitrogen).

### Electron microscopy sample preparation and analysis

Electron microscopy (EM) sample preparation and analysis were performed following previously described protocols [30, 27]. Telomere-enriched DNA was first spread in the presence of benzalkonium chloride (BAC) using water as hypophase. Formamide was used as a partially denaturing reagent to disentangle DNA molecules. In short, 5 μl of DNA solution corresponding to 5–20 ng of telomere-enriched DNA were mixed with 5 μl of formamide (Thermo Scientific) and 0.4 μl of 0.02% BAC (Sigma). After mixing, the drop was spread on a water surface in a 15-cm dish containing 50 ml of distilled water using a freshly cleaved mica sheet as a ramp. The monomolecular DNA film was then gently touched with carbon-coated EtBr-treated EM grids. The grids with absorbed DNA were immediately stained with 0.2 μg/μl uranyl acetate, washed in EtOH 100%, and subjected to platinum rotary shadowing. The detailed procedures of EM grid preparation and platinum rotary shadowing were described in reference [30]. Images were taken using FEI Tecnai12 BioTwin transmission electron microscope using the same setting as in references [27, 31].

EM images were obtained in large areas by acquiring overlapping fields and stitching them using the digital micrograph software. Images in .dm3 format from treated and untreated samples were randomized in ImageJ using the Blind Analysis Tools plugin and analyzed using an ImageJ macro for annotation and storage as in reference [27]. The analysis consisted in the annotation of the number of molecules containing i-loops among all the molecules present in the acquired area. The ratio between i-loops containing molecules over all the acquired molecules for each sample was reported and compared between the untreated and menadione-treated samples.

### Subcellular fractionation

Subcellular fractionation was performed using the Subcellular Protein Fractionation Kit for Cultured Cells (Thermo Fisher Scientific) according to the manufacturer’s protocol with some minor modifications. 10^7^ cells equivalent to 50 μl packed cell volume were used per condition. Cytoplasmic extract, membrane extract, nucleoplasmic extract, and pellet extract were collected following the manufacturer’s protocol. For chromatin-bound extract, the pellet was resuspended in 250 μl in NEB buffer at room temperature containing protease inhibitors, CaCl_2_, micrococcal nuclease, MgCl_2_, and benzonase endonuclease. The mixture was homogenized by passing through a 0.9 × 40 mm needle 10 times, vortexed for 15 s, and incubated at 37°C for 30 min. The sample was again vortexed for 15 s and centrifuged at 16 000× *g* for 5 min. The supernatant was collected as chromatin-bound extract.

### Western blotting

For whole cell lysate (WCL) samples, 0.5 × 10^6^ cells were lysed with 50 μl of radioimmunoprecipitation assay (RIPA) buffer (50 mM Tris–HCl, pH 8.0, 1% NP-40, 0.5% Na-deoxycholate, 0.1% SDS, 150 mM NaCl) supplemented with 250 U/ml benzonase and 2 mM MgCl_2_, and then diluted 1:1 with 2× Laemmli buffer.

The samples were boiled at 95°C for 10 min, followed by separation on a 4%–15% SDS–polyacrylamide gel electrophoresis (PAGE) precast gel (Mini-PROTEAN TGX Gels, Bio-Rad). The proteins were transferred onto 0.2 μm nitrocellulose membranes (Amersham) at 100 V for 90 min or 30 V for 16 h. The membranes were blocked with blocking solution [5% BSA (*w*/*v*) in 1× PBST (1× PBS + 0.1% Tween-20)] for at least 1 h, followed by overnight incubation at 4°C with primary antibodies against proteins of interest (see Supplementary Table S3) diluted in blocking solution. The membrane was then washed three times with PBST for 5 min per wash and incubated with horseradish peroxidase-conjugated secondary antibodies (see Supplementary Table S3) diluted in blocking solution for 1 h at room temperature. The membrane was again washed three times with PBST for 5 min per wash and developed using ChemiGlow Chemiluminescence Substrate (Bio Techne). The signal was detected with the Fusion FX imaging system (Vilber).

### Chromatin immunoprecipitation

10^7^ cells were crosslinked with 1 ml of 1% methanol-free formaldehyde (Thermo Fisher Scientific) diluted in 1× PBS for 15 min at room temperature on a rotating wheel. Formaldehyde was quenched with 200 mM Tris–HCl, pH 8.0 for 10 min on a wheel at room temperature. Cells were collected by centrifugation and washed with ice-cold PBS three times. The samples were flash-frozen and stored at −80°C, or used immediately.

Cells were lysed with 1 ml of lysis buffer (50 mM Tris–HCl, pH 8, 10 mM EDTA, 1% SDS) supplemented with protease inhibitor cocktail (cOmplete, Roche) for 10 min at room temperature, and then centrifuged at 3220× *g* for 5 min to collect chromatin sample. The chromatin pellet was washed once and resuspended with 1 ml of LB3 buffer (10 mM Tris, pH 8.0, 200 mM NaCl, 1 mM EDTA, 0.5 mM ethylene glycol-bis(β-aminoethyl ether)-*N*,*N*,*N*′,*N*′-tetraacetic acid (EGTA), 0.1% Na-deoxycholate, 0.25% sodium lauroyl sarkosinate) supplemented with protease inhibitor cocktail. The chromatin sample was then transferred to sonication vials with AFA fiber (Covaris) and fragmented using a Focused-Ultrasonicator (E220, Covaris) (10% duty factor, 140 W power, 200 cycles per burst, for 10 min) to achieve fragments between 200 and 500 bp. Sonicated chromatin was centrifuged at 4°C at 21 000x *g* for 15 min and the supernatant was collected. An amount equal to 5 μg of DNA (measured by NanoDrop spectrophotometer) was used for one immunoprecipitation (IP) with an appropriate amount (decided based on titration) of indicated antibodies (Supplementary Table S3) and 20 μl of 50% (*v*/*v*) protein G sepharose beads (Cytiva). The mixture was topped up to 1 ml per IP with IP buffer (50 mM Tris–HCl, pH 8.0, 600 mM NaCl, 10 mM EDTA, pH 8.0, 0.75% Triton X-100) supplemented with protease inhibitor cocktail and then rotated in cold room on a wheel overnight. An amount equal to 0.5 μg of DNA from each sample was kept as input.

The beads were collected by cold centrifugation at 400× *g* for 2 min the next day and washed at 4°C for 5 min per wash once with Wash 1 buffer (0.1% SDS, 1% Triton, 2 mM EDTA, pH 8.0, 20 mM Tris, pH 8.0, 300 mM NaCl), once with Wash 2 buffer (0.1% SDS, 1% Triton, 2 mM EDTA, pH 8.0, 20 mM Tris, pH 8.0, 500 mM NaCl), once with Wash 3 buffer (250 mM LiCl, 1% NP-40, 1% Na-deoxycholate, 1 mM EDTA, pH 8.0, 10 mM Tris, pH 8.0) and once with TE buffer. Input samples and the beads were resuspended in reverse-crosslink buffer (20 mM Tris–Cl, pH 8.0, 0.5 mM EDTA, 0.1% SDS, 0.1 M sodium bicarbonate) supplemented with 10 μg/ml DNase-free RNase (Roche) at 65°C on a rotating wheel overnight. DNA was isolated using the QIAquick PCR Purification kit (Qiagen), eluted in 100 μl H_2_O, and analyzed by dot blot.

### Dot blot analysis

Purified DNA was denatured at 95°C for 10 min and flash-cooled on ice. The samples were loaded onto Hybond-XL membranes (Amersham) and crosslinked with a Stratagene UV crosslinker at 254 nm. Membranes were denatured in 0.5 M NaOH, 1.5 M NaCl for 15 min, neutralized in 0.5 M Tris–Cl, pH 7.0, 1.5 M NaCl for 10 min with constant shaking at room temperature, pre-hybridized with Church buffer [0.5 M NaHPO_4_, 1 mM EDTA, pH 8.0, 1% (*w*/*v*) BSA, 7% SDS] for at least 1 h, and hybridized overnight with a C-rich telomere-specific probe at 65°C. After hybridization, the membranes were washed three times with wash buffer (2× SSC + 0.1% SDS) for at least 30 min per wash at 65°C and then exposed to a phosphor imager screen. The screen was scanned using a Typhoon imager (GE). Dot blot signals were quantified using AIDA software.

### DNA–RNA immunoprecipitation

DNA–RNA immunoprecipitation (DRIP) experiments were performed according to our published protocol [32]. 10^7^ cells were used per condition. Purified DNA was analyzed by dot blot (see above) or quantitative polymerase chain reaction (qPCR) [32]. Forward and reverse primers for qPCR are listed in Supplementary Table S4.

### TERRA RT-qPCR

RNA was isolated using the NucleoSpin RNA Isolation kit (Macherey-Nagel) according to the manufacturer's protocol. Three DNase treatments—two on the column and one in solution—were performed.

RT-qPCR for TERRA was carried out following our previously described protocol [33], using SuperScript III reverse transcriptase (Invitrogen) and Power SYBR Green PCR master mix (Thermo Fisher Scientific) on an Applied Biosystems 7900HT fast real-time system. Melting curve analysis was also included. Relative expression levels were analyzed by normalization to GAPDH housekeeping messenger RNA (mRNA) and compared to the RT negative control samples. Forward and reverse primers are listed in Supplementary Table S5.

### Immunofluorescence and telomeric fluorescence *in situ* hybridization

Cells were grown on round coverslips. Coverslips were washed twice in 1× PBS, fixed with 4% paraformaldehyde for 10 min at room temperature, and washed twice in 1× PBS. Coverslips were then incubated in permeabilization solution (0.1% Triton X-100, 0.02% SDS in 1× PBS) for 5 min, pre-blocked in 2% BSA dissolved in 1× PBS for 10 min, and blocked for 1 h with blocking solution [10% (*v*/*v*) normal goat serum in 2% (*w*/*v*) BSA dissolved in PBS]. Coverslips were incubated in humified chambers with primary antibodies overnight at 4°C, washed three times for 5 min in 1× PBS containing 2% BSA, 0.1% (*v*/*v*) Triton-100, and incubated with secondary antibody for 30 min at room temperature (Supplementary Table S3). The samples were washed again, fixed with 4% paraformaldehyde for 5 min at room temperature, and then dehydrated consecutively with 70%, 95%, and 100% ethanol.

For fluorescence *in situ* hybridization (FISH), hybridization was done with 100 nM Cy3-[CCCTAA]_3_ PNA probe (PNA Bio) in 10 mM Tris–Cl, pH 7.4, 70% formamide, 0.5% blocking reagent (Roche). Twenty microlitres of hybridization mix were used per coverslip. Incubation was at 80°C for 3 min, followed by 3 h at room temperature in a humidified chamber. Slides were washed twice for 15 min with wash buffer 1 (10 mM Tris–Cl, pH 7.4, 70% formamide) and three times for 5 min with wash buffer 2 (0.1 M Tris, pH 7.4, 0.15 M NaCl, 0.08% Tween-20). DAPI was added to the second last wash at 0.1 μg/ml. Slides were dehydrated with 70%, 95%, and 100% ethanol, air-dried and mounted with Vectashield.

Images were acquired with Leica SP8 equipped with a 63X/1.40 oil objective

### Electrophoretic mobility shift assay

Double-stranded DNA and DNA:RNA hybrids were formed by annealing the corresponding oligonucleotides (Supplementary Table S6). For annealing, the oligonucleotides were incubated for 3 min at 95°C followed by slow-cooling to room temperature in 50 mM Tris–Cl, pH 8.0, 10 mM MgCl_2_. The oligonucleotides shown as bottom strands in Fig. 5H were labeled at their 5′ ends with ^32^P.

Recombinant in-house-purified TRF1 protein was serially diluted in reaction buffer [50 mM Tris–Cl, pH 8.0, 2 mM MgCl_2_, 150 mM KCl, 0.5 mM DTT, 10% (*v*/*v*) glycerol], and incubated with 5 nM oligonucleotide substrates for 30 min at room temperature. Two microlitre of 6× loading dye buffer (ThermoFisher) were added to each of the reactions. Samples were loaded onto 1% agarose gels and run 2.5 h at 7 v/cm at 4°C in 1× TBE. Gels were dried for 2.5 h at 50°C on top of a N + membrane (Cytiva) and a sheet of Whatman paper. After exposing to a phosphor imager screen overnight, the screen was scanned using a Typhoon imager (GE).

### Data analysis and illustrations

IF-FISH images were processed and analyzed with ImageJ (2.14.0/1.54f). Dot blots were analyzed using Aida Image Analyzer (v.5.1). Preparation of graphs and statistical analyses was performed using GraphPad Prism (v. 10.4.1 (532). Two-tailed *t*-tests were used for comparison of two groups, whereas analyses of variance with Tukey’s multiple comparison test were applied for comparisons between three or more groups. Illustrations were created using BioRender and Adobe Illustrator.

## Results

### Oxidative stress induces accumulation of ssDNA breaks on both DNA strands at telomeres

**Figure 1.**
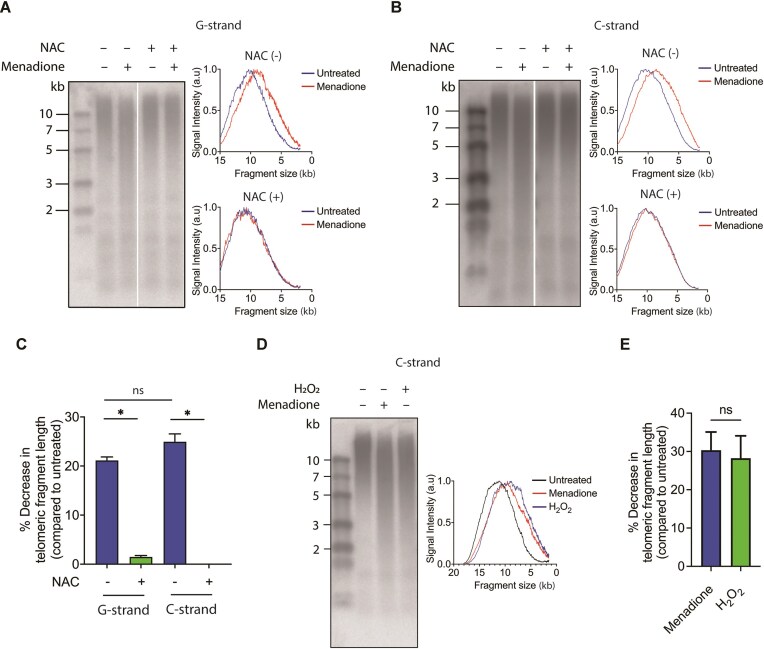
Accumulation of DNA SSBs at telomeres upon oxidative stress induction in HEK293E cells. Detection of telomeric SSB accumulation at G-rich (**A**) and C-rich (**B**) strands by alkaline gel electrophoresis upon menadione treatment with or without prior treatment with NAC. The signal intensity curves are shown on the right. (**C**) Quantification of DNA damage levels in panels (A) and (B), represented by the percentage decrease in telomeric fragment length of the menadione-treated sample compared to untreated. (**D**) Detection of telomeric SSB accumulation at C-rich strand by alkaline gel electrophoresis upon menadione and H_2_O_2_ treatment. The signal intensity curves are shown on the right. (**E**) Quantification of DNA damage levels in panel (D) represented by the percentage decrease in telomeric fragment length of the menadione-treated sample compared untreated. (*) *P* < .05, ns—not significant. Data represent mean ± SD. *n* = 3.

To study the effects of oxidative stress on telomere structure, we treated HEK293E cells with 0.1 mg/ml menadione for 2 h, which increases mitochondrial membrane permeability, leading to leakage of ROS into the cytoplasm and nucleus [34]. We isolated genomic DNA, digested it with frequently cutting restriction enzymes, which do not have recognition sites within the 5′-TTAGGG-3′ telomeric repeats. The digested DNA was analyzed using alkaline denaturing gels, which separate the two DNA strands, thereby allowing us to detect single DNA backbone cleavage events. The gels were hybridized with radiolabeled DNA probes specific for the telomeric G-rich or C-rich strand (Fig. 1A and B). We observed a shift in the signal intensity curve towards smaller fragments in the menadione-treated sample compared to the untreated one, indicating an accumulation of shortened single-stranded DNA (ssDNA) fragments caused by ROS-induced single strand break accumulation, in both the G-rich as well as C-rich strands. We also analyzed undigested DNA on alkaline denaturing gels. Ethidium bromide staining revealed smaller DNA fragments in menadione-treated cells indicating ssDNA breaks also elsewhere in the genome (Supplementary Fig. S1A), as seen before [5].

To confirm that the induction of DNA strand breaks by menadione was due to oxidative stress, we added 5 mM of the glutathione precursor NAC, which scavenges ROS [35], half an hour prior to adding menadione. NAC addition completely abolished the effects of menadione supporting the notion that menadione induced telomeric single-strand breaks through an increase of ROS (Fig. 1A–C). Consistently, treatment of cells with 10 mM H_2_O_2_ for 2 h had a similar effect on telomeric strands as menadione (Fig. 1D and E).

We also tested accumulation of DNA double-stranded breaks at telomeres upon menadione treatment by analyzing telomere length upon fractionation on native agarose gels and Southern hybridization (Supplementary Fig. S1B). Under these experimental conditions, the telomeric DNA fragment size did not decrease. Therefore, menadione did not elicit substantial amounts of double-stranded DNA breaks at telomeres, or they were not detected because of rapid repair [36].

**Figure 2.**
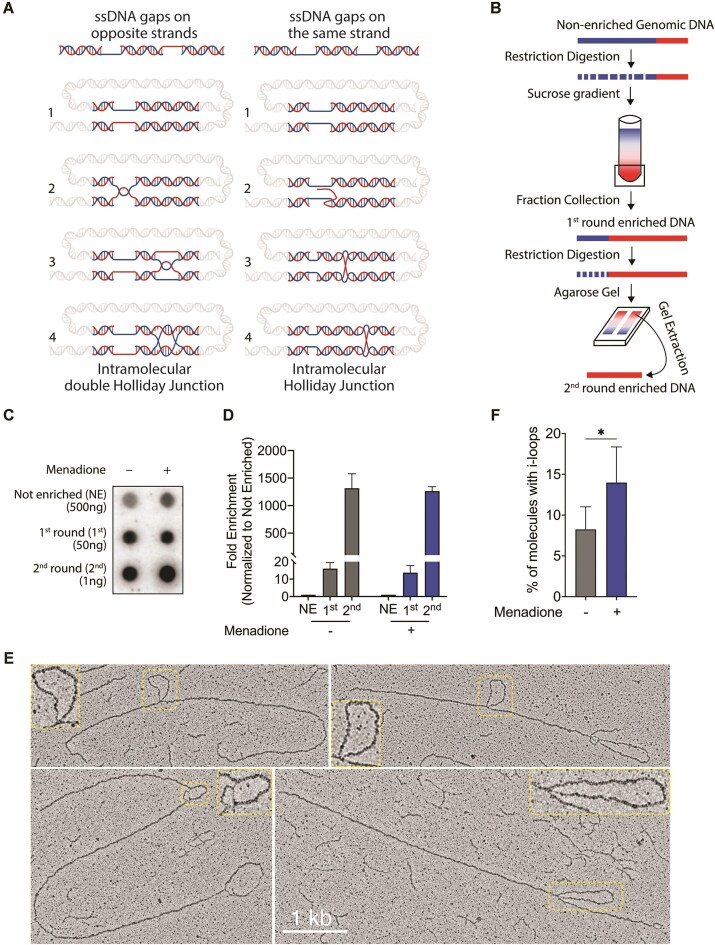
Accumulation of i-loop structures at telomeres upon oxidative stress induction in HEK293E cells. (**A**) Hypothetical models for i-loop structures induced by ssDNA gaps on telomeric strands (Adapted from [27]). This illustration was created in BioRender. Nguyen, T. (2025) https://BioRender.com/d44u613. (**B**) Schematic representation of the telomeric DNA enrichment procedure. (**C**) Dot blot analysis for quantification of the enrichment of telomeric repeats after the telomeric DNA purification procedure. The indicated amounts of DNA from each round of enrichment were blotted onto a membrane, which was then hybridized with telomere-specific probes. (**D**) Quantification of the telomeric DNA signal relative to the not-enriched DNA. (**E**) Representative EM images of telomeric DNA molecules with i-loops observed in the menadione-treated samples. (**F**) Percentages of molecules containing i-loops is determined based on the EM analysis in panel (E). (*) *P* < .05, ns—not significant. Data represent mean ± SD. *n* = 5.

Using electron microscopy (EM) analysis of purified telomeric DNA, it was recently discovered that telomeric repeats accumulate i-loops that occur in the proximity of nicks and ssDNA gaps [27]. I-loop structures may be formed by gaps present on the same or opposite telomeric strands, which can base-pair or facilitate strand invasion reactions as illustrated in Fig. 2A [27]. We tested if menadione treatment caused accumulation of telomeric i-loops. To preserve secondary DNA structures, the cells were treated with psoralen followed by exposure to 356-nm UV *in vivo*, which causes inter-strand crosslinks. Telomeric DNA was purified from total genomic DNA using a two-step procedure ([29]; Fig. 2B). Genomic DNA was digested with frequent cutting restriction enzymes lacking recognition sites in telomeric repeats. The digested DNA was first fractionated using a sucrose gradient to isolate high molecular weight telomeric DNA. This enriched telomeric DNA was then digested with another set of frequent cutters, and the resistant large telomeric fragments were further purified via agarose gel electrophoresis as a second step. The enrichment efficiency was measured by dot blot hybridization based on the telomeric DNA signal intensity per total amount of blotted DNA [29]. The two-step procedure resulted in more than a 1000-fold enrichment of telomeric DNA, suggesting near purity (Fig. 2C and D). The purified DNA was analyzed by EM for the presence of i-loops. Menadione treatment resulted in a nearly two-fold increase in i-loops with around 14% of the analyzed telomeric DNA molecules containing these structures (Fig. 2E and F). Consistent with previously the published data [27], menadione-induced i-loops mostly occurred in proximity of single-stranded gaps, which were apparent as thinner threads on the images (Fig. 2E). Overall, our data show that i-loop levels increase at telomeres following oxidative damage.

### ROS leads to TERRA upregulation and R-loop formation in *cis* and in trans

**Figure 3.**
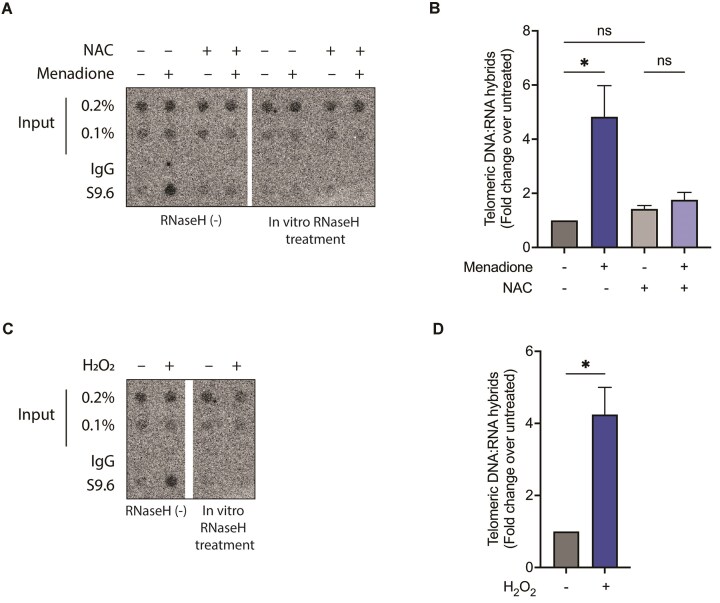
Upregulation of TERRA R-loops at damaged telomeres upon oxidative stress in HEK293E cells. Detection of total R-loops at telomeres upon menadione (**A**) or H_2_O_2_ (**C**) treatment by DRIP-dot blot assay using S9.6 antibody. *In vitro* digestion with RNaseH prior to the immunoprecipitation served as a control for R-loop specificity. Immunoprecipitated and input samples were blotted onto a membrane, followed by hybridization with a telomere-specific probe. (**B**, **D**) Quantification of telomeric DNA signal in (A) and (C), respectively, as fold change over untreated sample. (*) *P* < .05, ns—not significant. Data represent mean ± SD. *n* = 3

Genome-wide DNA damage promotes R-loop formation at damaged sites [37]. DNA damage and ROS have also been linked to R-loop formation at telomeres in ALT cancer cells, which contain strongly altered telomeric chromatin to maintain telomeres by recombination [20, 38–40]. We tested for the presence of R-loops at telomeres upon oxidative stress induction by menadione treatment in telomerase-positive HEK293E cells, which contain a canonical telomeric chromatin structure [41]. We performed DRIP in which the structure-specific antibody S9.6 was employed to precipitate R-loops from total nucleic acid preparations [42]. Precipitated nucleic acid was blotted onto a membrane, followed by hybridization with a C-rich telomere-specific probe (Fig. 3A and B). As a control for specificity, isolated nucleic acid was treated *in vitro* with RNaseH prior to immunoprecipitation. RNaseH specifically hydrolyses the RNA component of R-loops. Abolishment of the signal to background levels upon RNaseH treatment confirmed the specificity of this assay for telomeric R-loop detection. The DRIP data indicated that menadione leads to a five-fold increase in R-loops at telomeres in HEK293E cells (Fig. 3A and B). Preincubation with NAC prevented the accumulation of R-loops, demonstrating that the effect of menadione was mediated by ROS. Consistent with this conclusion, incubation of HEK293E cells with 10 mM H_2_O_2_ induced a similar increase in telomeric R-loops (Fig. 3C and D).

**Figure 4.**
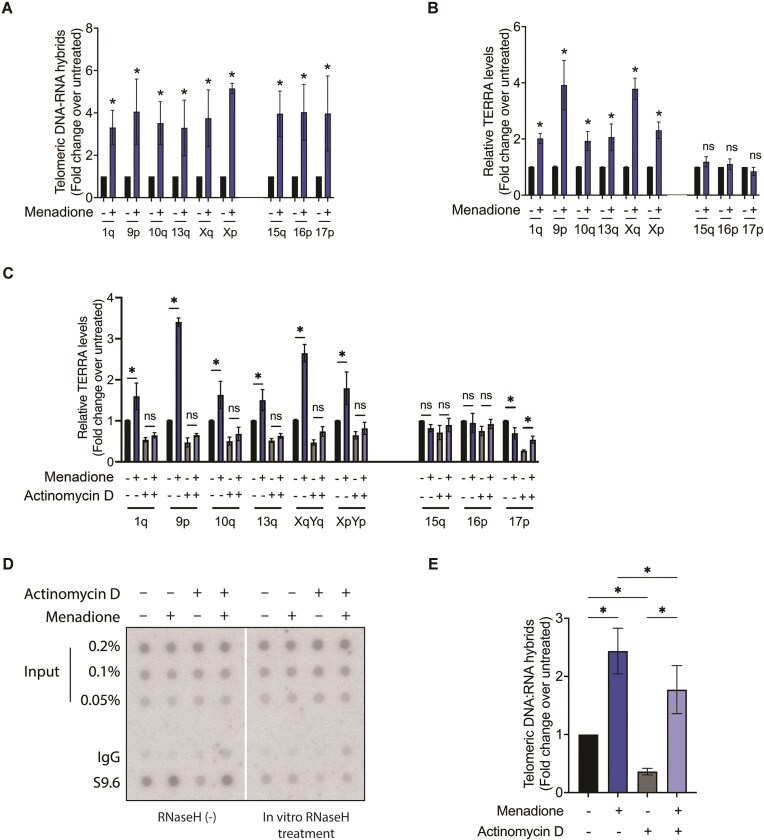
TERRA R-loops are formed both in-*cis* and in-trans at damaged telomeres upon oxidative stress in HEK293E cells. (**A**) R-loop levels at indicated chromosome ends measured by DRIP–qPCR upon menadione treatment. qPCR was performed using primers binding to chromosome-specific subtelomeric sequences. Data were calculated as percent of input, and shown as fold change over untreated sample. (**B**) Detection of TERRA transcribed from indicated chromosome ends upon menadione treatment by RT-qPCR, which was performed using primers binding to chromosome-specific subtelomeric sequences. Data were normalized to GAPDH, and are shown as fold change over untreated sample. (**C**) Detection of TERRA transcribed from indicated chromosome ends by RT-qPCR. The cells were treated with actinomycin D for 30 min, followed by 2 h of menadione treatment. Data are shown as fold change over untreated sample. (**D**) Detection of total R-loop level by DRIP-dot blot assay. The cells were treated with actinomycin D for 30 min, followed by 2 h of menadione treatment. (**E**) Quantification of telomeric DNA signal in panel (D) as fold change over untreated sample. (*) *P* < .05, ns—not significant. Data represent mean ± SD. *n* = 3

We also measured R-loops located at individual telomeres upon menadione treatment. Therefore, we used the DRIP samples from above (Fig. 3) for qPCR analysis using subtelomere-specific primers binding to sequences in close proximity to the telomeric 5′-TTAGGG-3′ repeats [33]. This analysis indicated that telomeric R-loop levels increased approximately four-fold at all nine tested chromosome ends upon menadione treatment (Fig. 4A). The increase in R-loop formation could happen in *cis* upon increased TERRA transcription, or in trans due to TERRA invasion post-transcriptionally, or both. Therefore, we examined the levels of TERRA molecules transcribed from the nine chromosome ends with RT-qPCR using primers specific for different subtelomeric sequences. GAPDH mRNA was used as a reference for RNA normalization. We observed that upon oxidative stress induction by menadione, TERRA expression was upregulated only at six out of the nine tested chromosome ends (Fig. 4B). In contrast, R-loops were increased at all tested telomeres including the ones from which TERRA expression was not increased (Fig. 4A and B). This suggests that the increase in R-loops did not occur only in *cis* due to the upregulation in TERRA transcription but that TERRA was recruited in trans to oxidatively damaged telomeres. To substantiate this finding, we blocked transcription by treating the cells with 5 μg/ml actinomycin D for half an hour prior to menadione treatment and analyzed R-loop levels (Fig. 4C–E). Actinomycin D treatment alone reduced TERRA (Fig. 4C) as well as R-loop levels (Fig. 4D and E) two-fold, indicating that transcription was efficiently suppressed. Interestingly, however, menadione treatment led to elevated R-loop formation at telomeres even when transcription was inhibited with actinomycin D (Fig. 4D and E). The increase in R-loops in the absence of transcription confirms the post-transcriptional recruitment of TERRA to damaged telomeres upon induction of oxidative stress. Overall, our data indicate that oxidative stress upregulates TERRA transcription at a subset of chromosome ends, leading to an elevation in the level of R-loops at all chromosome ends. TERRA is recruited to oxidatively damaged telomeres also in transforming R-loop structures post transcription. However, we failed to identify promoter elements that might explain the differential expression of TERRA at different chromosome ends in response to ROS.

### TRF1 dissociates from ROS-damaged telomeres

**Figure 5.**
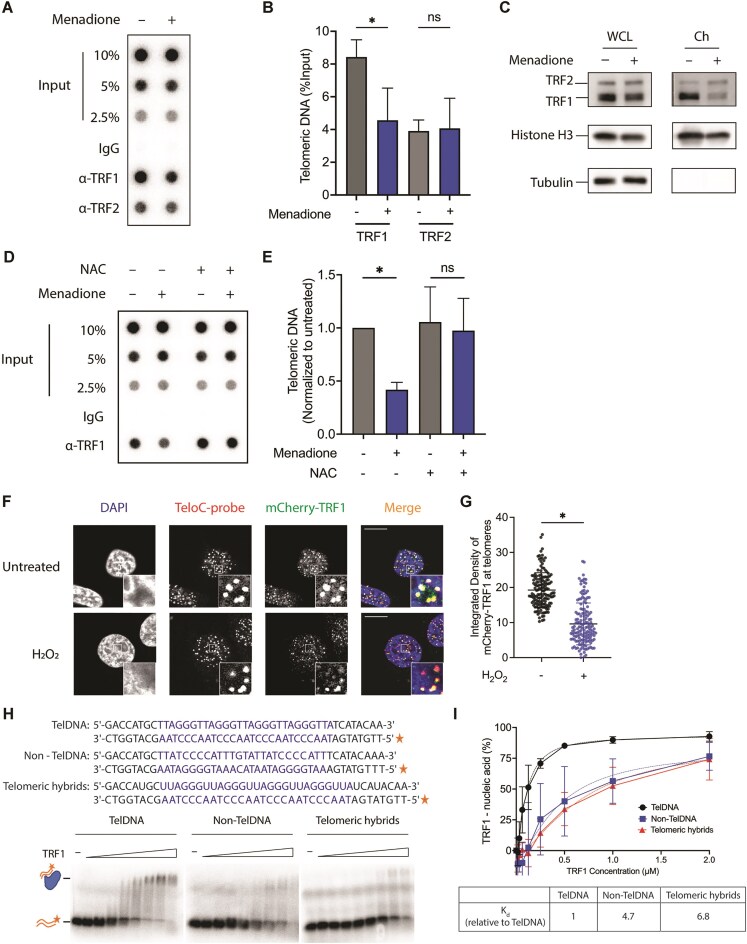
TRF1 proteins dissociate from oxidatively damaged telomeres in a ROS-dependent manner, while TRF2 proteins are unchanged. (**A**) Telomeric TRF1 and TRF2 protein levels detected by ChIP-dot blot following menadione treatment in HeLa cells. Immunoprecipitated DNA was blotted onto a membrane, which was then hybridized with a telomere-specific probe. (**B**) Quantification of telomeric DNA signal in panel (A) as percent of input. (**C**) Total WCL and chromatin-bound (Ch) levels of TRF1 and TRF2 proteins. Chromatin was extracted after menadione treatment using HEK293E cells containing endogenously FLAG-tagged TRF1 and TRF2 proteins. Western blot was done with an antibody against FLAG tag. Histone H3 and tubulin served as the loading control and as the fractionation control. (**D**) Telomeric TRF1 level detected by ChIP—dot blot following menadione treatment with or without prior NAC treatment in HeLa cells. (**E**) Quantification of telomeric DNA signal in panel (D) as fold change relative to untreated sample. (**F**) Representative figures of immunofluorescence (IF) of mCherry (green) and FISH of telomeric DNA (red), which were employed to assess the level of TRF1 protein at telomeres upon H_2_O_2_ treatment using HeLa cells containing endogenously mCherry-tagged TRF1. DAPI (blue) was used to stain nuclei. White squares show 15× zoom-in. Scale bar indicates 10 μm. (**G**) Average integrated density of mCherry foci colocalizing with telomeric DNA foci per nucleus. Each dot represents one nucleus. 150 cells were analyzed per condition, across three independent biological replicates. (**H**, **I**) The affinity of TRF1 for oligonucleotides containing non-telomeric dsDNA (non-TelDNA), telomeric dsDNA (TelDNA), and telomeric DNA:RNA hybrids (telomeric hybrids) was analyzed by electrophoretic mobility shift assay (EMSA). ^32^P-labelled (asterisks) oligonucleotides were incubated with increasing concentrations of TRF1 protein. The relative dissociation constants (*K*_d_) are shown in panel (I).

We assessed in cells the levels of TRF1 and TRF2 proteins at telomeres upon menadione treatment by chromatin immunoprecipitation (ChIP) using specific antibodies against both proteins. Precipitated telomeric DNA was measured by dot blot hybridization with a telomere-specific probe. The telomeric DNA signal obtained with anti-TRF1 antibody from the menadione-treated cells was reduced to 50% when compared to untreated cells, indicating that less TRF1 protein bound to oxidatively damaged telomeres (Fig. 5A and B). Pretreating the cells with the antioxidant NAC prior to menadione rescued TRF1 binding to telomeres, confirming that the reduced binding occurred in an oxidative-stress-dependent manner (Fig. 5A and B). In contrast to TRF1, the ChIP experiments with anti-TRF2 antibody did not reveal a reduction of TRF2 at oxidized telomeres (Fig. 5A and B). We also examined the telomeric levels of the shelterin protein components TPP1 and POT1 by ChIP and observed no significant changes following menadione treatment (Supplementary Fig. S2). To substantiate our observation, we fractionated cell lysates and measured TRF1 protein levels in WCLs and insoluble chromatin pellets. Histone H3 served as marker for chromatin fraction and tubulin for the WCL (Fig. 5C). We observed that there was markedly less TRF1 protein present in the chromatin fraction under oxidative stress conditions (Fig. 5C). In contrast, TRF2 levels did not diminish in the chromatin fraction upon menadione treatment. Furthermore, the TRF1 signal in the WCL was comparable between untreated and menadione-treated samples (Fig. 5C), indicating that the reduction of telomeric TRF1 protein was not due to a decrease in the total TRF1 protein level but due to changes in TRF1 localization. To confirm that the effects of menadione occurred via ROS, we again preincubated the cells with NAC prior to menadione addition. NAC pre-treatment completely rescued the TRF1 association with telomeres demonstrating that the effect of menadione was mediated by ROS (Fig. 5D and E).

To further corroborate our results, we combined immunofluorescence imaging with FISH in HeLa cells. TRF1 was endogenously tagged with mCherry and detected with an anti-mCherry antibody, and telomeric DNA was detected by FISH using a fluorescently labeled PNA probe (Fig. 5F and G). Oxidative stress was induced with H_2_O_2_. The intensity of mCherry-TRF1 foci colocalizing with telomeres was measured. The mCherry-TRF1 signal was reduced nearly two-fold in H_2_O_2_-treated cells consistent with the two-fold reduction of the telomeric ChIP signal obtained with anti-TRF1 antibodies.

Finally, we tested the possibility that increased DNA:RNA hybrid levels may be responsible for TRF1 dissociation from telomeres. We compared the binding affinity of recombinant purified TRF1 with double-stranded telomeric DNA and DNA:RNA hybrids by an EMSA (Fig. 5H and I). The affinity of TRF1 to telomeric DNA:RNA hybrids was approximately seven-fold lower when compared to double-stranded telomeric DNA. A non-telomeric double-stranded DNA sequence showed a five-fold reduction in binding. Thus, the data indicate that the non-B type DNA:RNA helices in R-loop structures disfavor TRF1 binding. The increase of telomeric R-loops in ROS-treated cells may explain the reduced presence of TRF1 at oxidized telomeres. However, the experiment does not rule out a contribution of oxidized guanine to TRF1-loss from telomeres. In previous work it had been demonstrated that base oxidation (8-oxo guanine) can disrupt the binding of TRF1 and TRF2 proteins to telomeric DNA oligonucleotide substrates *in vitro* [43]. However, since TRF2 was not reduced at telomeres in our experiments, it seems that the density of 8-oxo guanine was not elevated enough to impair its association.

### Increased R-loop formation requires TRF2

**Figure 6.**
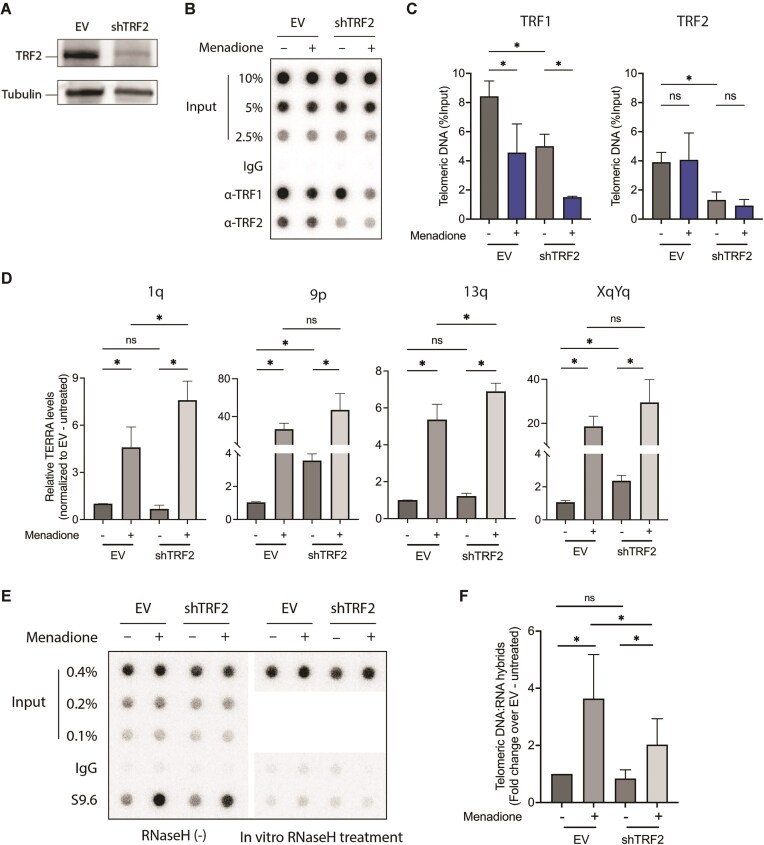
The upregulation of telomeric R-loops upon oxidative stress is TRF2-dependent. (**A**) Western blot analysis of TRF2 protein levels in HeLa cells transfected with empty vector (EV) or shTRF2 plasmids. Tubulin was used as a loading control. (**B**) Detection of telomeric TRF1 and TRF2 levels by ChIP-dot blot in wild-type (EV) and TRF2-knockdown (shTRF2) HeLa cells treated with menadione. Immunoprecipitated DNA was blotted onto a membrane, which was then hybridized with a telomere-specific probe. (**C**) Quantification of telomeric DNA signal in panel (B) as percent of input. (**D**) Levels of TERRA molecules transcribed from indicated chromosome ends upon menadione treatment were determined by RT-qPCR, using primers binding to chromosome-specific subtelomeric sequences. Data were normalized to GAPDH and shown as fold change relative to untreated sample. (**E**) Detection of total R-loop level by DRIP-dot blot assay upon menadione treatment in HeLa cells transfected with EV or shTRF2 plasmids. (**F**) Quantification of telomeric DNA signal in panel (E) as fold change over untreated sample. (*) *P* < .05, ns—not significant. Data represent mean ± SD. *n* = 3

As mentioned in the introduction, RAD51, RAD51AP1, and TRF2 have all been implicated to promote TERRA R-loop formation. A role of human RAD52 was also considered as it promotes annealing of complementary DNA strands [44]. TRF2 was a particularly interesting candidate as TRF1 represses its R-loop promoting activity in healthy cells [19]. To test their involvement, we depleted RAD51, RAD51AP1, RAD52, and TRF2 by RNA interference from untreated and menadione-treated HeLa cells (Supplementary Fig. S3 and Fig. 6A) and assessed telomeric R-loop formation by DRIP upon menadione treatment as in Figs 3 and 4. Depletion of RAD51, RAD51AP1, and RAD52 did not reveal a significant contribution of any of these factors for menadione-induced telomeric R-loop formation (Supplementary Fig. S3B and C). However, depletion of TRF2 showed a clear effect (Fig. 6). As expected, TRF2 depletion showed a reduced presence of this protein with telomeric DNA as assessed by ChIP (Fig. 6B and C). TRF2 depletion also slightly increased TERRA expression from a subset of telomeres consistent with published results [13] (Fig. 6D). Despite an increase in TERRA, TRF2 depletion showed by DRIP an approximately two-fold decrease in TERRA R-loops in menadione-treated cells but not in control cells (Fig. 6E and F). These results are consistent with a model in which the R-loop formation activity of TRF2 is inhibited in control cells by TRF1 but it becomes activated following TRF1 release upon oxidative damage. We conclude that TRF2 is critical for TERRA R-loop formation at ROS-damaged telomeres.

## Discussion

In this work, we increase oxidative stress by menadione treatment, which disrupts mitochondrial integrity and triggers elevated levels of intrinsic ROS throughout the cell [34]. Mitochondrial dysfunction is a hallmark of aging and typically associated with increased production of ROS [34]. Furthermore, the DNA damage response stemming from telomere dysfunction has been reported to compromise mitochondrial biogenesis and function, which leads to defective ATP generation and increased levels of ROS [45]. This may further increase the detrimental effects at telomeres in a positive-feedback loop [45]. In this perspective, our experimental setup mimics some of the processes associated with the aging (reviewed in reference [46]).

**Figure 7.**
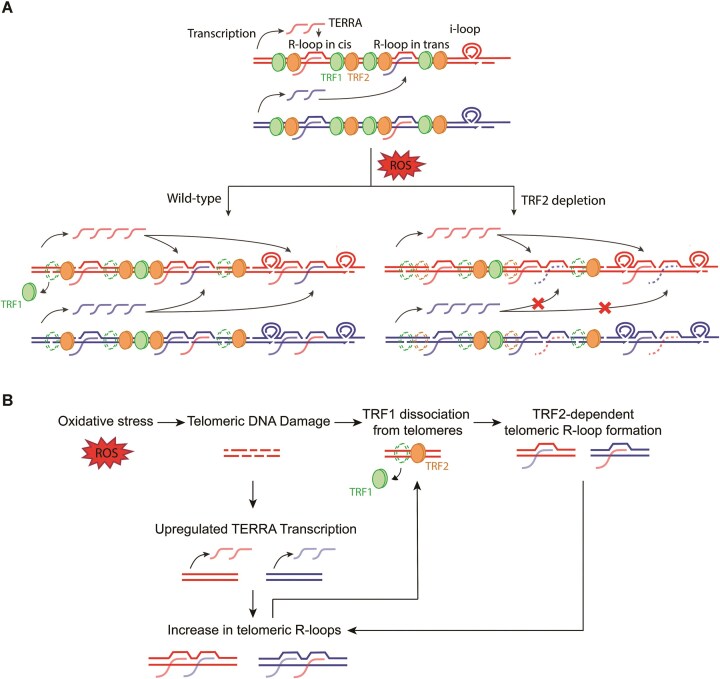
(**A**) Oxidative stress induces ssDNA damage, which may promote the formation of i-loops at telomeres. At damaged telomeres, TERRA transcription and recruitment are upregulated, leading to increases in R-loop formation both in *cis* and in trans. This enhanced TERRA recruitment and R-loop formation in trans may be due to the dissociation of TRF1 proteins from damaged telomeres, which alleviates TRF1’s inhibitory effect on TRF2-mediated TERRA invasion into telomeric DNA. (**B**) Hypothetical positive feedback model representing the potential interplay among TRF1, TRF2, and TERRA R-loops at oxidatively damaged telomeres.

Our results reveal that ROS has very strong impact on telomere structure (Fig. 7A). Oxidative stress leads to the accumulation of SSBs at both the G- and C-rich telomeric DNA strands. The SSBs can be generated either due to the processing of oxidized nucleotides by base excision repair or by direct backbone cleavage by ROS. Importantly, addition of the antioxidant NAC before menadione treatment suppressed telomeric damage demonstrating that menadione elicited its effects via ROS. We also find that ROS promotes formation of telomeric i-loops [27]. These structures may form adjacent to single-stranded gaps occurring at telomeric repeats and are particularly abundant in ALT cancer cells [27], which suffer from permanent DNA damage and are characterized by active homology directed repair. The i-loop structures can also be excised to generate extrachromosomal telomeric circles, which could potentially lead to telomere erosion [27].

Another major structural change induced by ROS is the strong increase in telomeric R-loop structures. The non-B forms of DNA:RNA hybrids have been demonstrated to be poor substrates for *in vitro* nucleosome assembly [47], and R-loops also profoundly affect histone deposition in live cells [48]. At ALT telomeres, deposition of the histone H3.3 variant by the histone chaperone HIRA counteracted telomeric R-loops suggesting that telomeric R-loops form at and possibly maintain an open chromatin structure [49]. Furthermore, we find that telomeric R-loop accumulation correlates with TRF1 dissociation from telomeres. Indeed, telomeric DNA:RNA hybrids largely withstand the binding of TRF1 *in vitro* consistent with published results [50]. Thus, R-loops in ROS-damaged telomeres may facilitate replacement of histones and canonical TRF1-containing shelterin complexes by DDR factors, which mediate damage signaling and repair. Interestingly, when double and ssDNA breaks were induced by ROS and DNA repair was investigated at a specific chromosomal locus, hybrids were only detected in the presence of local transcription suggesting that nascent RNA transcripts hybridize with DNA at sites of DNA damage facilitating recruitment of repair factors [51]. Our work at telomeres demonstrates that ROS-induced damage promotes R-loop formation at telomeres even upon inhibition of transcription by a post-transcriptional mechanism involving TRF2. Therefore, it seems that telomeres have evolved specialized mechanisms to promote R-loop formation and facilitate subsequent repair. A more robust mechanism of R-loop formation may have evolved at telomeres as they are prone to ROS-induced damage and as telomere damage is detrimental to chromosome stability and cell cycle progression for tissue renewal.

The increased R-loop formation at ROS-damaged telomeres may be sustained by several mechanisms. First, we observe that ROS induces TERRA expression from several chromosome ends. Second, the accumulation of ROS-induced ssDNA breaks may initiate R-loop formation. Indeed, *in vitro* data show that nicks in the non-template strand favor R-loop formation [52]. Third and most critical, we observe that R-loop formation upon ROS-induced damage requires TRF2 (Fig. 7). It is possible that R-loop promoting activity of TRF2 becomes unhinged upon TRF1 release from chromatin, as demonstrated previously by TRF1 depletion [19]. Thus, we propose a positive feedback loop in which subsequent to ROS-induced SSBs, the upregulation of TERRA facilitates R-loop formation and TRF1 release. TRF1 release in turn unleashes the TRF2 mediated promotion of TERRA R-loops, which triggers the release of further TRF1 molecules in a positive feedback loop (Fig. 7B). It will be important to further test this model in future work and fascinating to identify in a comprehensive manner the proteins that associate with ROS-damaged telomeres to facilitate telomeric DDR and repair.

## Supplementary Material

gkaf285_Supplemental_File

## Data Availability

The data underlying this article will be shared on reasonable request to the corresponding authors.

## Notes

Present address: 4bases Italia S.r.l., 27100 Pavia, Italy

Present address: German Cancer Research Center (DKFZ), 69120 Heidelberg, Germany

Present address: LifeMine Therapeutics, 4057 Basel, Switzerland
