# Supplementary material for: Oxidative stress at telomeres triggers internal DNA loops, TRF1 dissociation, and TRF2-dependent R-loops
Source: Nucleic Acids Res. 2025 Apr 12;53(7):gkaf285. doi: 10.1093/nar/gkaf285 (PMC11992676; doi:10.1093/nar/gkaf285)
Supplement: gkaf285_Supplemental_File [file gkaf285_supplemental_file.pdf]

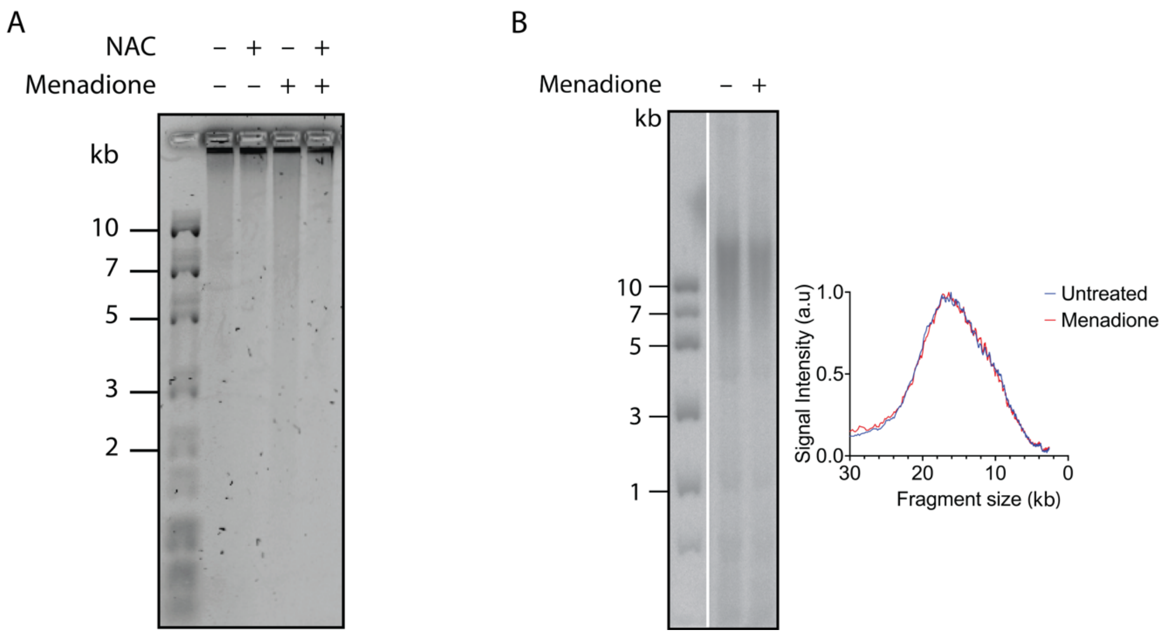

Supplementary Figure 1: (A) Menadione treatment causes genome-wide accumulation of DNA single-stranded breaks. Undigested genomic DNA was separated on an alkaline gel, which was then neutralized and stained with ethidium bromide. **(B)** Telomeric DNA double-stranded breaks are not detected upon menadione treatment. Native gel electrophoresis was performed to resolve telomeric fragments which were detected upon hybridization with a telomere-specific radiolabeled probe. The distribution of the telomeric DNA fragments is shown on the right by the signal intensity curves.

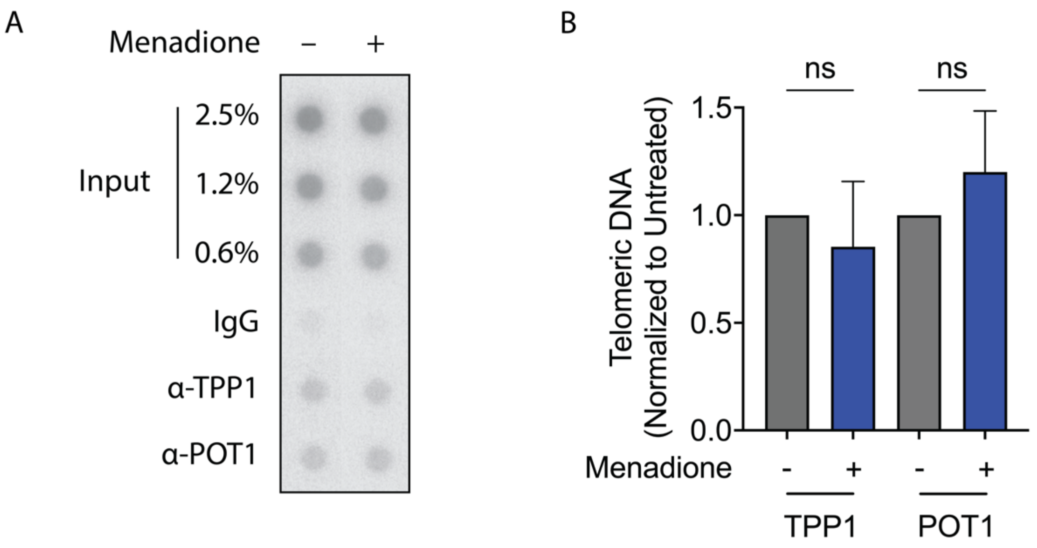

Supplementary Figure 2: TPP1 and POT1 telomeric DNA binding analyzed by ChIP is not affected by menadione treatment. (A) Telomeric DNA detected upon ChIP using TPP1 or POT1 antibodies following menadione treatment in HEK293E cells. Immunoprecipitated DNA was blotted onto a membrane which was then hybridized with a telomere-specific probe. **(B)** Quantification of telomeric DNA signals in **(A)** relative to untreated samples**.**
(*) $p < 0.05$, ns – not significant. Data represents mean ± SD. n = 3

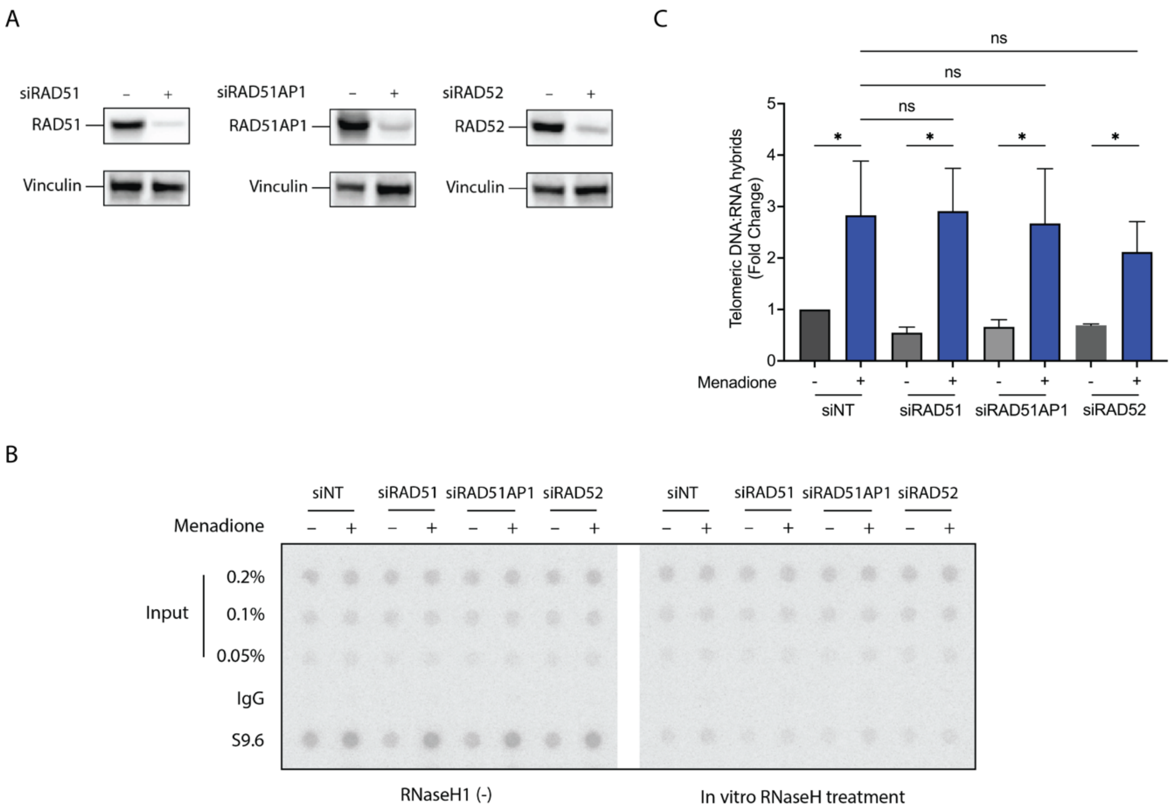

Supplementary Figure 3: The upregulation of TERRA R-loops upon oxidative stress is not affected by depletion of RAD51, RAD51AP1 or RAD52. (A) Western Blot analysis showing depletion efficiency with indicated siRNAs in Hela cells. Vinculin was used as a loading control. **(B)** Detection of total R-loop levels by DRIP-dot blot assay upon menadione treatment in Hela cells transfected with indicated siRNAs. **(C)** Quantification of telomeric DNA signal in **(B)** as fold change over untreated sample.

(*) $p < 0.05$, ns – not significant. Data represents mean ± SD. n = 3

Supplementary Table 1. siRNA information

siRNA	Information
shNon-targeting	Dharmacon: D-001810-10
siRAD51	Dharmacon: M-003530-04
siRAD51AP1	Ouyang et al., 2021<br>Sequence: GCAGUGUAGCCAGUGAUUATT
siRAD52	Dharmacon: L-011760-00

Supplementary Table 2. shRNA information

shRNA	Information
shEV	Negative control shRNA; backbone: pSUPER.Puro (OligoEngine)
shTRF2	shRNA for TRF2 depletion; backbone: pSUPER.Puro (OligoEngine); target sequence: GCGCATGACAATAAGCAGA

Supplementary table 3. Antibodies

Application	Antibody target	Supplier: catalog number	Dilution/Amount
Western Blot	FLAG	Sigma: A8592	1:2000
	Histone H3	Abcam: ab1791	1:10000
	RAD51	SantaCruz: sc-8349	1:2000
	RAD51AP1	ProteinTech: 11255-1-AP	1:2000
	RAD52	SantaCruz: sc-365341	1:1000
	Vinculin	Abcam: ab129002	1:10000
	Tubulin	SantaCruz: sc-8035	1:5000
	Mouse IgG (H+L) HRP-conjugated	Promega: W4021	1:10,000
	Rabbit IgG (H+L) HRP-conjugated	Promega: W4011	1:10,000
DRIP	DNA:RNA hybrid	Kerafast: ENH001	0.2 µg/µg nucleic acid
	Mouse IgG	SantaCruz: sc-2025	
ChIP	TRF1	In-house	Appropriate amount according to titration
	TRF2		
	Sheep IgG	ImmunoReagents	
IF - FISH	mCherry	Abcam: ab167453	1:100
	Alexa-488	ThermoFisher: 704060	1:1000

Supplementary Table 4. Primers for DRIP-qPCR

Target loci	Oligonucleotide Sequences (5’-3’)
1q Fwd	TAGTGTGGAAAGCGGGAAAC
1q Rev	TGCAGTTGAACCCTGCAATA
9p Fwd	GAGATTCTCCCAAGGCAAGG
9p Rev	ACATGAGGAATGTGGGTGTTAT
10q Fwd	GCATTCCTAATGCACACATGAC
10q Rev	TACCCGAACCTGAACCCTAA
13q Fwd	GCACTTGAACCCTGCAATACAG
13q Rev	CCTGCGCACCGAGATTCT
XqYq Fwd	AGCGTCGGAACGCAAAT
XqYq Rev	TGGGTATCATGTGTGCATTAGG
XpYp Fwd	CCACAACCCCACCAGAAAGA
XpYp Rev	GCGCGTCCGGAGTTTG
15q Fwd	TGCAACCGGGAAAGATTTTATT
15q Rev	GCGTGGCTTTGGGACAACT
16p Fwd	GCCTGGCTTTGGGACAACT
16p Rev	TGCAACCGGGAAAGATTTTATT
17p Fwd	CTTATCCACTTCTGTCCCAAGG
17p Rev	CCCAAAGTACACAAAGCAATCC

Supplementary Table 5. Primers for RT-qPCR

Target loci	Oligonucleotide Sequences (5’-3’)
1q Fwd	TAGTGTGGAAAGCGGGAAAC
1q Rev	TGCAGTTGAACCCTGCAATA
9p Fwd	TCCCTATAATCCGCCACTACT
9p Rev	ACATTGCAGGGTCCTCTTG
10q Fwd	GCCTTGCCTTGGGAGAATCT
10q Rev	AAAGCGGGAAACGAAAAGC
13q Fwd	GCACTTGAACCCTGCAATACAG
13q Rev	CCTGCGCACCGAGATTCT
XqYq Fwd	TCCTAATGCACACATGATACCC
XqYq Rev	CCCTAAGCACATGAGGAATGT
XpYp Fwd	GAGTGAAAGAACGAAGCTTCC
XpYp Rev	CCCTCTGAAAGTGGACCTAT
15q Fwd	TGCAACCGGGAAAGATTTTATT
15q Rev	GCGTGGCTTTGGGACAACT
16p Fwd	GCCTGGCTTTGGGACAACT
16p Rev	TGCAACCGGGAAAGATTTTATT
17p Fwd	GGGACAGAAGTGGATAAGCTGATC
17p Rev	GATCCCACTGTTTTTATTACTGTTCCT

Supplementary Table 6. EMSA oligonucleotide sequence

Name	Oligonucleotide Sequences (5'-3')
TelDNA-sense	GACCATGCTTAGGGTTAGGGTTAGGGTTAGGGTTATCATACAA
TelDNA-antisense	TTGTATGATAACCCTAACCCTAACCCTAACCCTAAGCATGGTC
TelRNA-sense	GACCAUGCUUAGGGUUAGGGUUAGGGUUAGGGUUAUCAUACAA
Non-TelDNA-sense	GACCATGCTTATCCCCATTTGTATTATCCCCATTTCATACAAA
Non-TelDNA-antisense	TTTGTATGAAATGGGGATAATACAAATGGGGATAAGCATGGTC
